# RGB Image-Based Lightweight Eggplant Fruit Disease Recognition via Frequency Spatial Collaborative Downsampling and Dynamic Attention

**DOI:** 10.3390/s26216690

**Published:** 2026-10-22

**Authors:** Shijie Mo, Chenglin Wen

**Affiliations:** 1 College of Computer Science, Guangdong University of Petrochemical Technology, Maoming 525000, China; mosj@gdupt.edu.cn; 2 Guangdong Provincial Key Laboratory of Petrochemical Equipment Fault Diagnosis, Guangdong University of Petrochemical Technology, Maoming 525000, China

**Keywords:** eggplant fruit disease recognition, field images, downsampling, attention mechanism, lightweight convolutional networks, smart agriculture

## Abstract

Eggplant is an important vegetable crop, and accurate identification of fruit diseases is critical for ensuring yield and quality. However, field-captured eggplant images may contain small disease lesions, foliage occlusion, and illumination variations, posing challenges for accurate disease recognition. To address these challenges, this paper proposes E-GhostNetV3 (Eggplant-GhostNetV3), a lightweight disease recognition network for field-captured images. The core improvements include a frequency–spatial collaborative downsampling module (WCD-Down), which combines Haar-based multiband decomposition with parallel convolution for spatial feature extraction, and a Dynamic Spatial–Channel Attention (DySCA) module to improve spatial and channel-wise feature representation. Evaluated on an eggplant disease dataset comprising 1823 real field-captured images across five classes, E-GhostNetV3 achieves 89.61% Top-1 accuracy with 1.81 M parameters and an estimated 116.60 M MACs under the default THOP counting rules, outperforming the GhostNetV3 baseline (87.07%) by 2.54 percentage points. These results demonstrate the effectiveness of E-GhostNetV3 on the evaluated dataset and its potential as a lightweight model for edge-based smart agriculture applications. The study also provides a benchmark for evaluating models trained from scratch under data-scarce conditions.

## 1. Introduction

Eggplant (*Solanum melongena* L.) is an important vegetable crop widely cultivated around the world, with particularly significant economic value in Southeast Asia. However, eggplant fruits are susceptible to various diseases, including Shoot and Fruit Borer, Wet Rot, Brinjal Fruit Cracking, and Phomopsis Blight, which not only affect fruit quality but also cause substantial economic losses. Traditional disease diagnosis mainly relies on manual visual inspection, which is time-consuming, labor-intensive, and highly subjective, making it difficult to meet the demands of large-scale precision agriculture. Recently, with the widespread availability of RGB cameras and smartphone cameras, large-scale field image acquisition has become feasible [1]. Nevertheless, images captured in complex field environments may be affected by illumination variations, foliage occlusion, and cluttered backgrounds, posing challenges for deep learning-based disease recognition. Therefore, developing automated, high-throughput recognition systems that are tailored to the characteristics of field-captured RGB images is of great significance for smart agriculture.

Before the widespread adoption of deep learning, plant disease recognition primarily relied on handcrafted features such as color, texture, and shape, combined with traditional classifiers like Support Vector Machines (SVM). Barbedo [2] conducted a systematic review of plant disease detection methods based on visible-light images, pointing out that traditional image processing approaches suffer from issues such as sensitivity to illumination and background interference under field conditions, resulting in limited generalization capability. Although such methods perform reasonably well on small-scale datasets collected under controlled conditions, their performance may be affected by variations in illumination, background complexity, and symptom appearance, which can limit their generalization to real-world field scenarios. With the advent of deep learning, particularly the widespread application of Convolutional Neural Networks (CNNs), image-based automatic plant disease recognition has made significant progress. CNNs are capable of automatically learning multi-level semantic features from large-scale image data, generally outperforming traditional handcrafted feature methods in lesion texture modeling, boundary capture, and background suppression. Yousha et al. [3] (2025) provided a systematic review of deep learning methods in eggplant disease detection, covering various CNN model architectures, image processing techniques, and data sources, and concluded that CNNs are among the most effective techniques for eggplant disease detection. To address the challenges of lightweight design and feature enhancement on resource-constrained devices, researchers have proposed various network improvements based on attention mechanisms. For instance, Sharma et al. proposed the AELGNet architecture, which integrates residual channel attention and spatial attention for collaborative modeling [4].

Despite considerable progress, existing studies still face several challenges. First, many publicly available plant disease datasets have been collected under laboratory or controlled conditions, often featuring relatively uniform backgrounds and stable lighting, which may limit the generalization of models to real-world field environments. Second, repeated downsampling in conventional CNN backbones may reduce spatial resolution and lead to the loss of fine-grained details, potentially limiting the representation of small lesions. Third, under field conditions, low contrast between small lesions and complex backgrounds may make it difficult for lightweight networks to distinguish discriminative lesion features from background textures. These challenges motivate further investigation into feature-preserving architectures and adaptive feature refinement strategies for field-based disease recognition.

Recent studies have increasingly explored lightweight deep learning models to improve the efficiency and robustness of crop disease recognition and detection in complex agricultural environments. For example, EggplantDet integrates a lightweight architecture with a dedicated small-object detection strategy to improve the identification of small-scale and early-stage disease symptoms while reducing computational costs [5]. Similarly, SSANet combines lightweight feature extraction with multi-scale adaptive feature recalibration to enhance local detail representation and suppress background interference in dynamic agricultural scenarios [6]. These studies demonstrate the potential of lightweight architectures to achieve a favorable balance between recognition performance, computational efficiency, and robustness under challenging field conditions. Nevertheless, further improvements are needed in preserving fine-grained lesion information and effectively modeling discriminative features when disease symptoms are small, low-contrast, or embedded in complex backgrounds.

Lightweight architectures such as MobileNetV3 and EfficientNet are designed to balance feature representation capability and computational efficiency through architectural strategies such as depthwise separable convolutions, inverted residual blocks, and efficient feature scaling. Although these designs reduce computational costs, their effectiveness in fine-grained disease recognition may depend on the characteristics of the input images and the distribution of discriminative features. In particular, depthwise separable convolutions reduce computational cost by separating spatial filtering from channel-wise feature mixing. While computationally efficient, this design may impose different feature interaction patterns from standard convolutions, whose implications for subtle lesion discrimination warrant further investigation. In eggplant fruit disease images, some symptoms appear as small lesions with subtle differences in texture, color, and boundary patterns. Repeated spatial downsampling may reduce the resolution of these local patterns, making it more challenging to preserve subtle lesion-related information in deeper feature representations. In addition, conventional convolutional architectures primarily learn spatial features through convolutional operations and do not explicitly decompose feature maps into frequency subbands. Consequently, complementary frequency-domain information may not be explicitly exploited during feature extraction. These considerations motivate the exploration of feature-preserving downsampling and complementary spatial-frequency representations for eggplant fruit disease recognition. Furthermore, attention mechanisms that emphasize channel-wise or spatial feature responses may provide additional feature refinement, although their effectiveness can depend on the complexity of the background and the characteristics of the target lesions. Therefore, improving the representation of fine-grained lesion features while maintaining computational efficiency remains an important consideration in lightweight eggplant fruit disease recognition.

These challenges are relevant to eggplant fruit disease recognition, yet dedicated research in this area has started relatively late and suffers from significant limitations. Existing studies on eggplant diseases have predominantly focused on leaf disease recognition. Krishnaswamy and Purushothaman [7] an early publicly reported eggplant leaf disease dataset, achieving 99.4% accuracy under controlled conditions using VGG16 combined with MSVM. However, this study only addressed leaf diseases and relied on image acquisition under laboratory conditions. In contrast, fruit disease recognition presents distinct challenges, and although publicly available datasets exist, their class coverage is limited. The BrinjalFruitX dataset released by Hasan et al. [8] extends the publicly available eggplant fruit disease datasets to five classes (including healthy fruits and four common diseases), providing a more comprehensive benchmark for eggplant fruit disease recognition research. However, systematic studies on lightweight recognition models specifically for eggplant fruit diseases remain scarce. Based on this dataset, Nath et al. [9] conducted a systematic benchmarking of multiple CNN architectures and found that MobileNetV2 achieved 97.98% accuracy when using ImageNet pre-trained weights. However, they also found that the performance of all models dropped significantly without pre-trained weights, indicating that training from scratch on this dataset remains a challenging setting. This paper therefore focuses on exploring whether targeted architectural improvements can enhance lightweight model performance under training-from-scratch conditions, without relying on external pretrained weights. This focus does not imply that transfer learning is unsuitable or inferior; rather, it defines the training setting investigated in the present study. Evaluating models trained from scratch provides insight into their ability to learn feature representations without relying on large-scale external pre-training data, which is relevant to agricultural applications where access to suitable pre-trained models or large-scale datasets may be limited.

Rather than pursuing a fundamentally novel architecture, we focus on incremental yet targeted improvements to a strong lightweight baseline (GhostNetV3 [10]), introducing two plug-in modules—WCD-Down and DySCA—that are specifically designed to address the information loss during downsampling and the enhancement of discriminative power across multi-stage features.

Specifically, the WCD-Down module introduces a parallelized restructuring of the original Stem stage of GhostNetV3. Recent work by Finder et al. [11] has demonstrated the potential of integrating wavelet transform (WT) into CNNs. Inspired by this, the module retains the original standard convolution branch while adding a parallel Haar discrete wavelet transform branch to extract multi-band structural information from the input image. The feature maps from the two branches are concatenated along the channel dimension and then passed through a fusion convolution layer for information integration and spatial downsampling. The rationale behind this design is that standard convolution captures local spatial patterns, while the two-dimensional Haar discrete wavelet transform decomposes input features into four frequency subbands, namely LL, LH, HL, and HH. The LL subband primarily represents low-frequency structural information, whereas the LH, HL, and HH subbands represent high-frequency variations in different orientations, including edge and fine-texture information. This multiband representation provides a theoretical motivation for retaining complementary low- and high-frequency information during feature downsampling. The parallel complementary nature of the two branches is intended to reduce the risk of losing or blurring discriminative features (e.g., lesion boundaries) during single-branch downsampling. However, because the Haar transform uses fixed decomposition filters, it cannot adapt these filters directly to dataset-specific characteristics. The learnable convolution branch therefore provides data-driven spatial feature extraction to complement the fixed frequency-domain representation, while the subsequent fusion convolution enables the network to learn how to integrate the two types of features. Furthermore, with the aim of improving feature representation in field images, inspired by the Dynamic Spatial Attention (DSA) proposed by Xiong Z et al. [12], we insert a Dynamic Spatial–Channel Attention (DySCA) module after Stages 1 and 2 of the shallow network of GhostNetV3. DySCA is an incremental improvement over DSA: it retains the dynamic spatial attention while adding dynamic channel attention. Meanwhile, it extends the statistics used in spatial attention from a single mean to a comprehensive combination of mean, maximum, and standard deviation, enabling more comprehensive capture of spatial distribution characteristics. By performing collaborative adaptive reweighting across spatial and channel dimensions on intermediate feature maps, the design aims to guide the network to focus on lesion regions in the spatial domain while enhancing responses to discriminative feature channels in the channel domain, thereby aiming to improve the representation of discriminative features, including those associated with small lesions.

Specifically, the main contributions of this paper are as follows:

- Incremental lightweight improvement: We propose E-GhostNetV3 (Eggplant-GhostNetV3) based on GhostNetV3, incorporating WCD-Down and DySCA to enhance the representation of fine-grained lesion features in field images. The model improves recognition accuracy while retaining a compact parameter count and is designed with the computational constraints of mobile and edge devices in mind.
- WCD-Down downsampling module: This module restructures the Stem stage of GhostNetV3 by retaining the original standard convolution branch while adding a parallel Haar discrete wavelet transform branch. Through concatenation and fusion convolution, it is designed to provide complementary spatial and frequency-domain representations and mitigate potential detail loss during downsampling.
- DySCA dynamic spatial–channel attention module: Inspired by the DSA of Xiong Z et al. [12], we design the DySCA module, which builds upon dynamic spatial attention by (1) extending the single-mean statistic to a comprehensive combination of mean, maximum, and standard deviation, and (2) incorporating dynamic channel attention. This forms a spatial–channel collaborative adaptive reweighting mechanism, inserted after Stages 1 and 2 of the shallow network of GhostNetV3, aiming to enhance the model’s responsiveness to discriminative features.
- Comprehensive evaluation: Experiments are conducted on a real field-collected eggplant fruit disease dataset, including comparisons with multiple lightweight baselines, ablation studies, and confusion matrix analysis, demonstrating the effectiveness of the proposed method on the evaluated dataset and its potential for future edge-based agricultural applications.

The remainder of this paper is organized as follows: Section 2 details the materials and methods; Section 3 reports the experimental results and analysis; Section 4 presents the discussion; and Section 5 summarizes the work and outlines future directions.

## 2. Materials and Methods

### 2.1. BrinjalFruitX Dataset

The eggplant fruit disease dataset (BrinjalFruitX) used in this study was released by Hasan et al. [8], comprising 1823 RGB images covering five classes representing healthy fruits and four common disease infections. All images were collected in actual agricultural fields from two major eggplant-growing regions in Bangladesh—Bogura and Dhaka, captured using standard smartphone cameras under natural lighting conditions, without any artificial modification or background control during acquisition. Initially, 2273 raw images were collected, and after rigorous quality screening, annotation, and expert validation, 1823 images were ultimately confirmed. However, the released BrinjalFruitX dataset does not provide reliable image-level metadata linking individual images to their specific collection locations. Therefore, the two regions cannot be used to construct a strictly location-disjoint training and test split based on the available dataset information.

Each class exhibits distinct visual symptoms: Shoot and Fruit Borer presents as boreholes on the fruit surface with surrounding brown discoloration; Wet Rot causes fruit softening and decay, with water-soaked brown patches on the surface; Brinjal Fruit Cracking manifests as longitudinal cracks on the pericarp, sometimes accompanied by tissue necrosis at the crack margins; and Phomopsis Blight appears as sunken spots on the fruit surface. Among these, Wet Rot and Phomopsis Blight share similar appearances at the late stage of rot, and this visual similarity provides a pathological basis for the inter-class confusion observed in the subsequent confusion matrix analysis.

The distribution of the five classes is shown in Table 1. Among them, the Shoot and Fruit Borer and Healthy classes have relatively larger sample sizes, whereas Phomopsis Blight has fewer samples. This class distribution reflects the composition of the collected field dataset rather than an artificially balanced sampling strategy. Therefore, the original class distribution was retained during the stratified dataset split.

All images are stored in JPEG format with varying original resolutions and were uniformly resized to 224×224 pixels prior to experiments. Pixel values were then normalized to the [0, 1] range and standardized using the ImageNet mean and standard deviation. The images were captured using smartphones under natural sunlight in outdoor field scenes, with each image manually annotated and validated by experts in the agricultural domain.

The dataset was split at the image level using a stratified 8:1:1 ratio, which roughly maintains the class distribution among the training, validation, and test subsets. Specifically, approximately 80% of the images from each class were used for training, 10% for validation, and the remaining 10% for testing. All compared methods were trained and evaluated using the same split. The splitting unit was an individual image rather than a plant, fruit, acquisition session, or geographic location. The available BrinjalFruitX dataset does not provide image-level metadata linking individual images to specific fruits, plants, acquisition sessions, or locations. Therefore, plant-level, fruit-level, acquisition-session-level, or location-level separation cannot be reliably performed based on the available dataset information. Furthermore, the image-level split used in this study cannot fully rule out correlations between subsets arising from images of the same fruit or similar acquisition scenes. Given the class imbalance, no class-specific resampling or class-balancing strategy was applied at the dataset-sampling level, so that the same data distribution was maintained for all compared models.

### 2.2. Modeling Methodology

#### 2.2.1. E-GhostNetV3 Network Architecture

This paper proposes an improved lightweight network architecture, E-GhostNetV3 (Eggplant-GhostNetV3), building upon GhostNetV3 [10] as the backbone, aiming to enhance classification accuracy for eggplant fruit disease recognition while reducing the parameter count relative to the original GhostNetV3 baseline.

**Figure 1:**
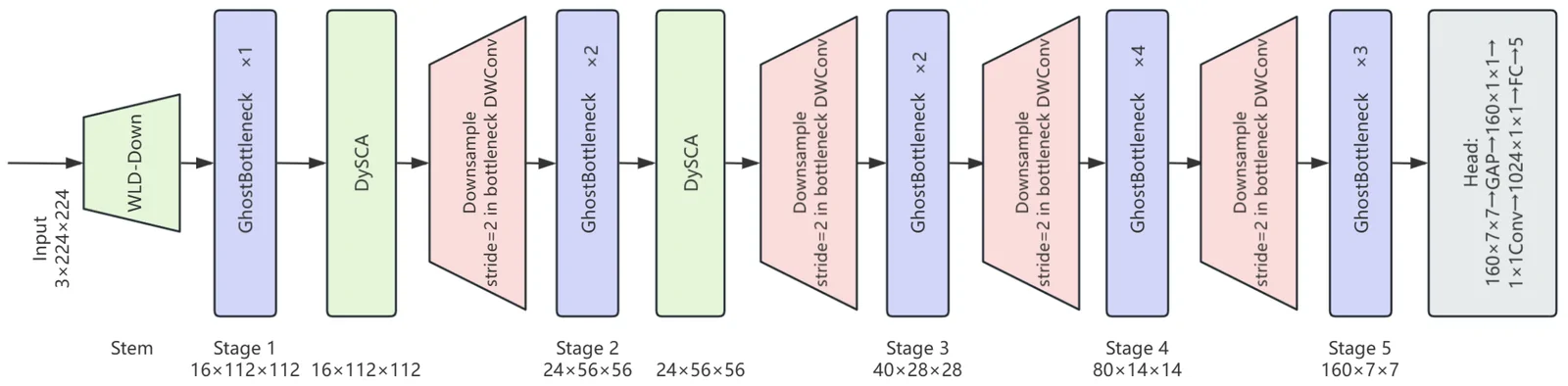
Schematic diagram of the proposed E-GhostNetV3 architecture.

E-GhostNetV3 adopts GhostNetV3 as the backbone. The network consists of a convolutional stem, five feature extraction stages, and a classification head. The input RGB images are first passed through the WCD-Down module for downsampling and preliminary feature extraction to obtain enhanced shallow features. They then sequentially go through Stages 1 to 5, where the spatial resolution gradually decreases and the channel numbers progressively increase to capture multi-scale semantic information. After the final stage, global average pooling and a 1×1 convolution compress the representations into a 1280-dimensional feature vector, which is fed into a fully connected layer to produce predictions for the five eggplant disease classes. As shown in Figure 1, the overall architecture of E-GhostNetV3 integrates these components in a streamlined manner.

One of the core improvements of E-GhostNetV3 is the replacement of the original standard convolutional downsampling in the Stem stage with the WCD-Down module. This module retains the original standard convolution branch and adds a parallel Haar discrete wavelet transform branch, which performs spatial-domain feature extraction and frequency-domain multi-band decomposition on the input image respectively. The output feature maps from the two branches are concatenated along the channel dimension and then integrated through a fusion convolution for information integration and spatial downsampling. This design aims to mitigate the loss of discriminative features during downsampling through a frequency-spatial-domain collaborative feature extraction and fusion strategy. Furthermore, inspired by the Dynamic Spatial Attention (DSA) of Xiong Z et al. [12], we insert the DySCA (Dynamic Spatial–Channel Attention) module after Stages 1 and 2 of the shallow network. This module performs collaborative adaptive reweighting on intermediate feature maps along both spatial and channel dimensions, with the design intention of strengthening the network’s perception of small lesions.

Although the Haar wavelet transform provides an explicit frequency-domain decomposition, its filters are fixed and therefore cannot adapt their decomposition characteristics directly to the dataset. To address this limitation, WCD-Down does not rely exclusively on the wavelet branch. Instead, the Haar-based branch is combined with a learnable strided convolution branch, which retains the ability to adaptively extract spatial patterns and local textures from the input data. The outputs of the two branches are concatenated and subsequently fused using a 1×1 convolution, enabling the network to learn how to integrate the fixed frequency-domain representation with the adaptive spatial features. In this way, the fixed Haar transform serves as an explicit frequency-domain prior, while the learnable convolution and subsequent network layers provide data-dependent feature adaptation.

As summarized in Table 2, the reconstruction retains the configurations of Stages 1 and 2, while the main structural compression is introduced in the later stages. In Stage 3, the block configuration and expansion/output dimensions are retained, but the SE module is removed. In Stage 4, the number of blocks is reduced from six to four by removing the two blocks with 112 output channels. In Stage 5, the number of blocks is reduced from five to three, and the intermediate channel dimensions are reduced from 672/960/960/960/960 to 256/512/768, while the output channel dimension remains 160. These structural modifications substantially reduce the model complexity. The reconstructed backbone contains 2.18 M parameters when SE modules are retained and 1.80 M parameters when SE modules are removed.

The effect of these modifications is examined in the ablation study presented later in this paper. The reconstructed backbone with SE achieves 84.64% accuracy, compared with 87.07% for the original GhostNetV3, quantifying the performance trade-off introduced by the backbone reconfiguration. Removing SE from the reconstructed backbone increases the accuracy to 86.63% while further reducing the parameter count to 1.80 M. The SE-free reconstructed backbone is therefore adopted as the common baseline for the subsequent WCD-Down and DySCA experiments, allowing the contributions of the proposed modules to be evaluated under the same lightweight backbone configuration.

#### 2.2.2. WCD-Down Module

In field disease recognition, lesion regions typically occupy only a small portion of the image. The indiscriminate downsampling of standard convolutions may cause the structural information of small lesions to be diluted by background pixels during receptive field expansion, while fine details at lesion boundaries are easily discarded in stride operations. To address this issue, we design the WCD-Down (Wavelet Conv Downsampling) module as a replacement for the standard convolution downsampling in the Stem stage of the GhostNetV3 backbone. The concept of this module is inspired by the WTConv of Finder et al. [11], but is specifically adapted for the Stem stage of GhostNetV3.

Specifically, this module employs 2D discrete Haar wavelet transform to decompose the input feature maps, as the work of Finder et al. [11] has validated the effectiveness of Haar wavelet transform for multi-frequency analysis and feature extraction in CNNs. Unlike Finder et al., who aimed to replace standard convolutions with WTConv layers to enlarge receptive fields, we apply wavelet transform to the shallow downsampling stage of the network, with the core objective of preserving frequency-domain structural information to compensate for the detail loss caused by standard convolution downsampling, with the aim of retaining discriminative information during downsampling.

**Figure 2:**
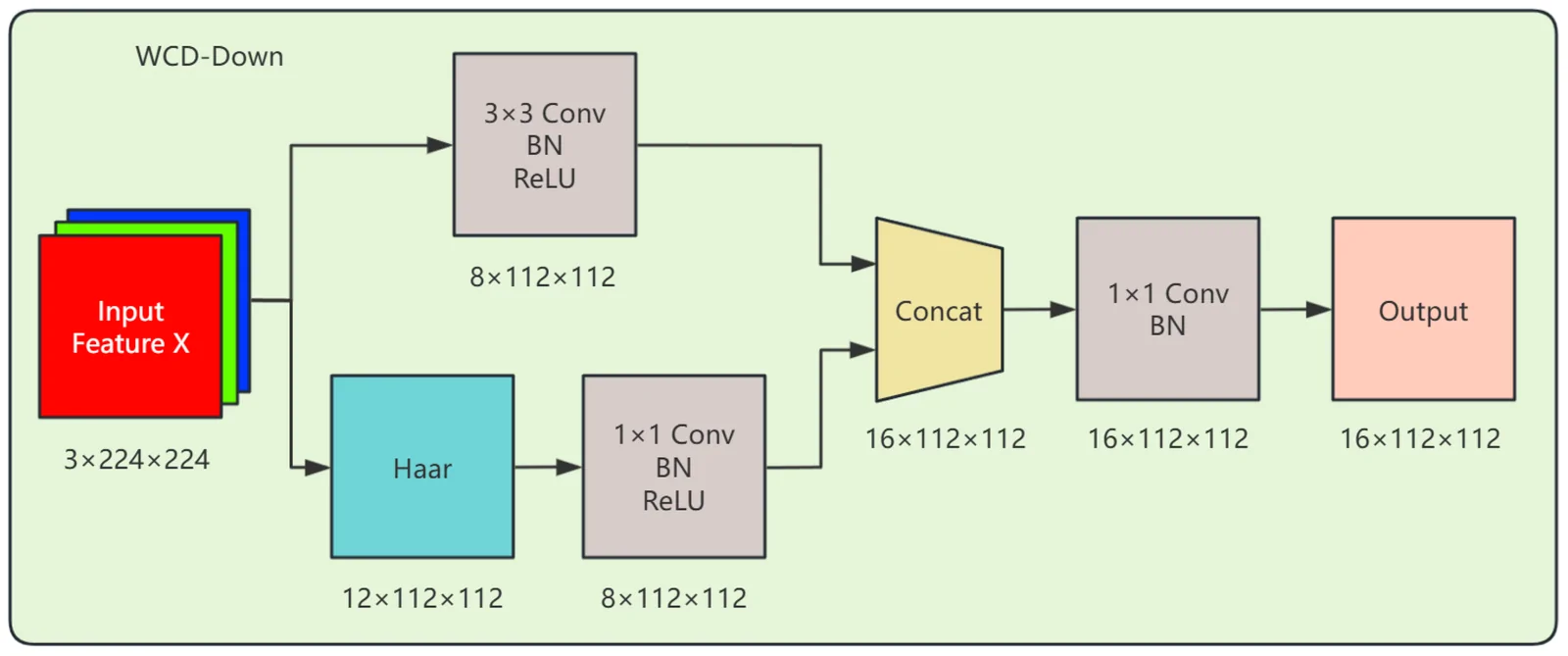
Internal architecture of the WCD-Down downsampling module used in E-GhostNetV3.

Let the input feature map be $X\in R^{C\times H\times W}$ (for the network input layer, C=3, H=W=224), WCD-Down consists of two parallel branches, as illustrated in Figure 2:

For the standard convolution branch, a 3×3 standard convolution with stride 2, batch normalization (BN), and ReLU activation are first applied to the input *X*, performing spatial downsampling and channel expansion, yielding the output $X_{conv}$:

(1) $$X_{conv}=\mathrm{ReLU}\left(\mathrm{BN}\left({\mathrm{Conv}}_{3\times 3,\mathrm{stride}=2}\left(X\right)\right)\right)\in R^{C^{\prime }\times H/2\times W/2},$$

where $C^{\prime }=\frac{C_{out}}{2}$ is half of the output channel number, and $C_{out}=16$ in the configuration shown in Figure 2.

For the frequency-domain feature extraction branch, 2D discrete Haar wavelet transform is first applied to the input *X* along the spatial dimensions (see Finder et al. [11] for detailed computation), decomposing the image into four frequency subbands: the low-frequency approximation subband $X_{LL}\in R^{C\times H/2\times W/2}$, and three high-frequency detail subbands $X_{LH},X_{HL},X_{HH}\in R^{C\times H/2\times W/2}$. These four subbands are concatenated along the channel dimension to obtain $X_{cat}\in R^{4C\times H/2\times W/2}$, which contains the main structural information (low-frequency) as well as local texture and edge variations (high-frequency) of the image. Subsequently, $X_{cat}$ is passed through a 1×1 standard convolution with stride 1 for channel transformation, batch normalization, and ReLU activation, yielding the frequency branch output $X_{freq}$:

(2) $$X_{freq}=\mathrm{ReLU}\left(\mathrm{BN}\left({\mathrm{Conv}}_{1\times 1}\left(X_{cat}\right)\right)\right)\in R^{C^{\prime }\times H/2\times W/2}.$$

The outputs of the two branches are then concatenated along the channel dimension and fused through a 1×1 convolution and batch normalization to integrate spatial-domain and frequency-domain features, producing the fused feature $X_{fuse}$:

(3) $$X_{fuse}=\mathrm{BN}\left({\mathrm{Conv}}_{1\times 1}\left(\mathrm{Concat}\left(X_{conv},X_{freq}\right)\right)\right)\in R^{2C^{\prime }\times H/2\times W/2}.$$

This design enables the downsampled features to retain both the spatial-domain texture details and the frequency-domain structural priors, providing richer feature information for subsequent feature extraction and attention generation.

#### 2.2.3. DySCA Module

**Figure 3:**
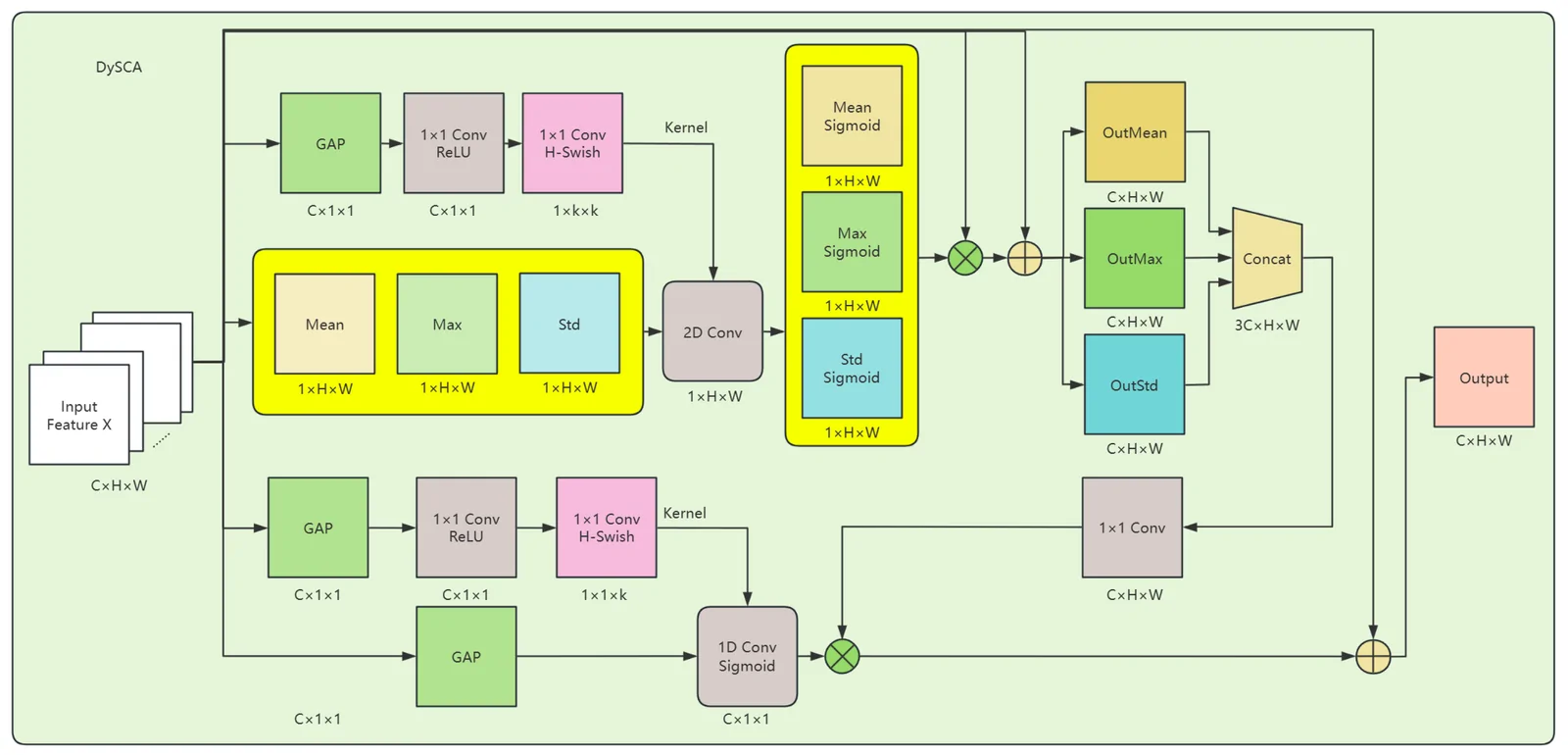
Internal architecture of the DySCA module used in E-GhostNetV3.

Let the input feature map be $X\in R^{C\times H\times W}$. The DySCA module consists of two components—spatial attention and channel attention—which perform adaptive reweighting on the feature map along the spatial and channel dimensions, respectively. The overall structure of the module is illustrated in Figure 3.

For spatial attention, a dynamic spatial convolution kernel ${\mathrm{Kernel}}_{s}=\Phi \left(X\right)\in R^{1\times 5\times 5}$ is first generated based on the input *X*, with a kernel size of 5×5. The generation function Φ is defined as:

(4) $$\Phi \left(X\right)=\mathrm{HSwish}({\mathrm{Conv}}_{1\times 1}\left(\mathrm{ReLU}\left({\mathrm{Conv}}_{1\times 1}\left(\mathrm{GAP}\left(X\right)\right)\right)\right)).$$

Inspired by the DSA of Xiong Z et al. [12], the generation philosophy of this dynamic convolution kernel is consistent with DSA, producing sample-specific kernel parameters based on the input *X*. Subsequently, three spatial descriptors—$X_{mean}$, $X_{max}$ and $X_{std}\in R^{1\times H\times W}$—are obtained by computing the mean, maximum, and standard deviation of the input *X* along the channel dimension. Unlike DSA, which uses only the mean, DySCA additionally introduces the maximum and standard deviation to capture spatial distribution characteristics more comprehensively—the maximum highlights local extreme regions, while the standard deviation reflects local dispersion, both of which help emphasize the contrast between lesions and the background. These three descriptors are respectively convolved with the dynamically generated ${\mathrm{Kernel}}_{s}$, passed through a Sigmoid function to generate attention weights, and then element-wise multiplied by the original feature *X* to obtain three sets of attention-enhanced feature maps, which are then added back to the original input *X* in the form of residual connections:

(5) $$\begin{array}{cc} X_{mean}^{\prime } & =\sigma ({\mathrm{Conv}}_{5\times 5}\left(X_{mean}\right))⊙X+X,\\ X_{max}^{\prime } & =\sigma ({\mathrm{Conv}}_{5\times 5}\left(X_{max}\right))⊙X+X,\\ X_{std}^{\prime } & =\sigma ({\mathrm{Conv}}_{5\times 5}\left(X_{std}\right))⊙X+X, \end{array}$$

where ⊙ denotes element-wise multiplication and σ denotes the Sigmoid activation function. The Conv5×5 operation is shown in Figure 3. It convolves $X_{mean}$, $X_{max}$, and $X_{std}$ with the same dynamic convolution kernel generated by Φ(X). The three outputs are concatenated along the channel dimension and fused through a 1×1 convolution, yielding the fused output of the spatial attention branch:

(6) $$X_{s}={\mathrm{Conv}}_{1\times 1}\left(\mathrm{Concat}\left(X_{mean}^{\prime },X_{max}^{\prime },X_{std}^{\prime }\right)\right)\in R^{C\times H\times W}.$$

As an extension of the dynamic spatial attention design philosophy, we further introduce dynamic channel attention to form a spatial–channel dual collaborative mechanism. Unlike spatial attention, which operates on the spatial dimensions (H×W), channel attention computes attention weights along the channel dimension (*C*). A channel convolution ${\mathrm{Kernel}}_{c}=\Phi \left(X\right)\in R^{1\times 1\times 5}$ is first generated based on the input *X* through the same Φ function as the spatial branch. Subsequently, global average pooling is applied to *X* to obtain the channel descriptor:

(7) $$X_{pool}=\mathrm{GAP}\left(X\right)\in R^{C\times 1\times 1},$$

Treating $X_{pool}$ as a 1D sequence of length *C*, a 1D convolution with ${\mathrm{Kernel}}_{c}$ (a 1×5 convolution kernel) is performed along the channel dimension. The result is passed through a Sigmoid function to generate channel attention weights, with the weight shape being C×1×1, which are then applied to the spatial attention output $X_{s}$, with a residual connection added back to the original input *X*:

(8) $$X_{out}=\sigma \left({\mathrm{Conv}}_{1\times 5}\left(X_{pool}\right)\right)⊙X_{s}+X,$$

where ${\mathrm{Conv}}_{1\times 5}$ denotes a 1D convolution along the channel dimension with a kernel size of 5. The final $X_{out}\in R^{C\times H\times W}$ is the output of the DySCA module.

#### 2.2.4. Positioning and Comparison of WCD-Down/DySCA

Table 3 provides a structured comparison of WCD-Down and DySCA with representative modules across four practical dimensions (Core Mechanism, Placement, Advantages and Limitations).

In this paper, WCD-Down is deployed at the Stem stage to replace the original standard convolutional downsampling, with the aim of mitigating potential information loss during spatial resolution reduction. The reconstructed backbone removes the original SE modules, while DySCA incorporates both spatial and channel attention and is inserted after Stages 1 and 2. This shallow placement is motivated by the relatively high spatial resolution and local spatial details of early feature maps, which may support attention-based feature modeling. In contrast, deeper feature maps have lower spatial resolution and more abstract semantic representations. The benefits of extending attention to deeper stages may depend on feature characteristics and network configuration. Therefore, deeper placement does not necessarily yield additional performance gains.

### 2.3. Training Settings

All experiments are conducted under the same training environment and hyperparameter settings to ensure fair comparison. All models are implemented using Python 3.11.14 and the PyTorch 2.10.0 deep learning framework (CUDA 13.0), and are trained and evaluated on a single NVIDIA GeForce RTX 3090 GPU (NVIDIA Corporation, Santa Clara, CA, USA). The main training parameters are summarized in Table 4.

To ensure a controlled comparison, all baseline models and E-GhostNetV3 were trained using the same loss function (soft-target cross-entropy), cosine learning-rate scheduler, data augmentation, and sampling strategy. The applied augmentations, including horizontal flipping, color perturbation, Mixup (0.1), and CutMix (0.3), were used consistently across all models rather than being specifically designed to rebalance the minority class. Similarly, no class-weighted loss, Focal Loss, or class-specific resampling strategy was introduced. These settings were kept consistent across all models to ensure that performance differences were evaluated under the same training and data-processing conditions, without introducing additional imbalance-handling mechanisms that could affect the comparison.

Model size is reported using the number of parameters (Params). Computational complexity is estimated using the default counting rules of THOP 0.1.1 with an input tensor of size 1×3×224×224, and the resulting values are reported as MACs estimates rather than FLOPs. No custom counting rules were added. Supported standard layers within custom modules are counted, but functional dynamic convolutions and other unsupported operations are not fully covered. The estimates therefore do not represent a complete accounting of all model operations. Although the same profiling tool and input size were used for all models, counting coverage may differ across architectures. These estimates do not measure inference latency, throughput, or energy consumption.

It should be noted that the training of all models in this paper differs fundamentally from existing studies on the BrinjalFruitX dataset [9]. Nath et al. [9] all employed ImageNet pre-trained weights for transfer learning or fine-tuning, whereas all models in this paper are trained from scratch without using any external pre-trained weights. This choice prevents direct numerical comparison of our experimental results with those studies, but constitutes an important focus of this work—demonstrating the effectiveness of the proposed WCD-Down and DySCA modules in improving the feature learning capability of lightweight models under the more challenging “no prior knowledge” condition, which provides important reference value for agricultural application scenarios lacking large-scale pre-training data.

To account for run-to-run variability, each experiment was repeated five times with different random initializations. The random seeds used were as follows: 42, 43, 44, 45, 46. The mean and 95% confidence intervals (95% CIs) are reported to characterize the variability observed across the repeated runs.

**Figure 4:**
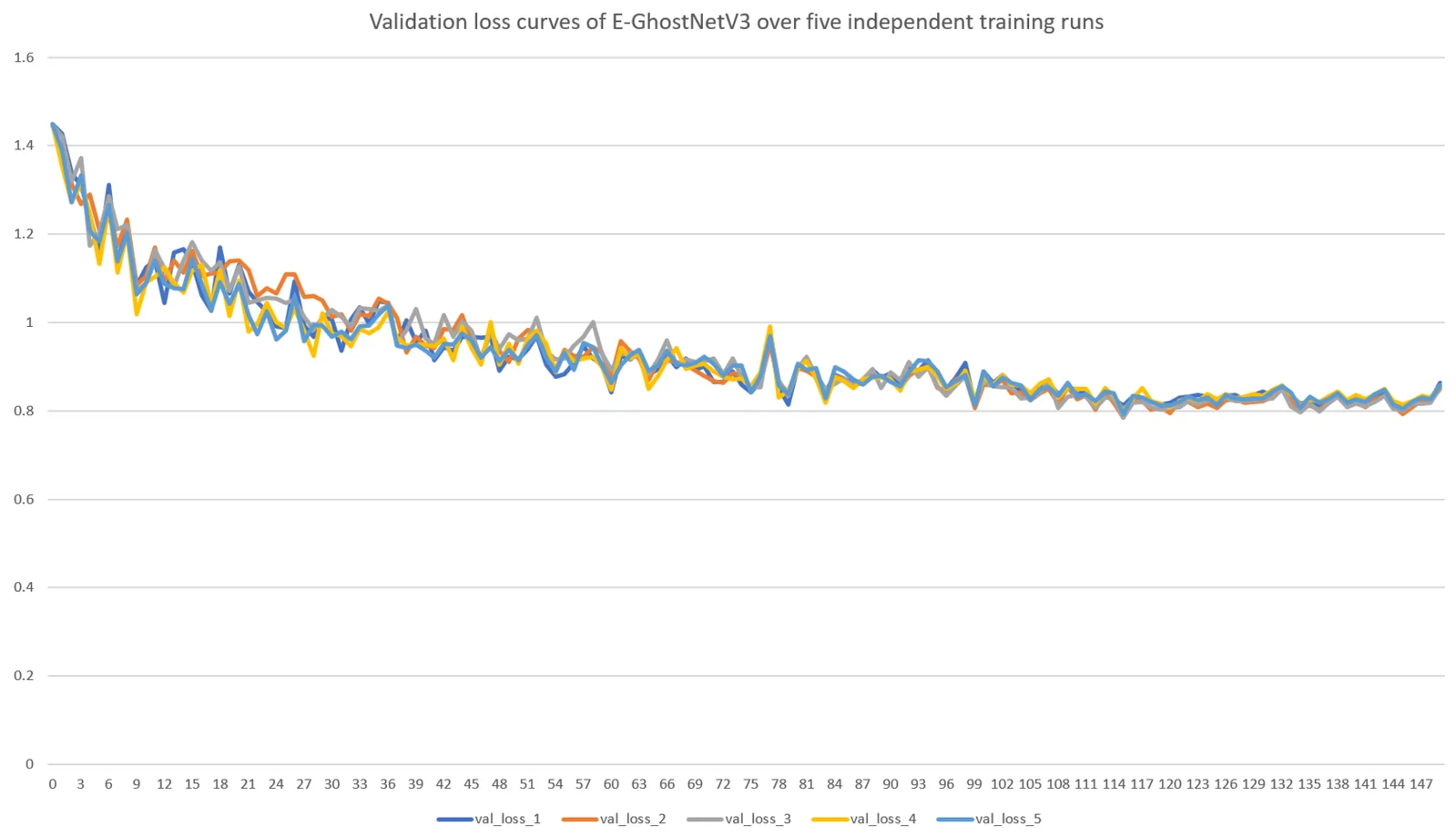
Validation loss curves of E-GhostNetV3 over five independent training runs.

All models were trained from scratch for 150 epochs. To assess the adequacy of the training duration, the validation loss was recorded at each epoch for all five independent runs of E-GhostNetV3. As shown in Figure 4, the validation loss decreases substantially during the early and middle stages of training and enters a relatively stable range after approximately 110–120 epochs across the five runs, with only moderate fluctuations thereafter. This observed training behavior provides empirical support for the selected 150-epoch training schedule, which was adopted for all models to ensure a consistent and controlled comparison. For each run, the checkpoint with the highest validation accuracy was used for final evaluation.

To evaluate classification performance, we adopt Precision, Recall, Accuracy, and F1-score as evaluation metrics. For multi-class evaluation, we employ a one-vs-rest strategy, where each class is treated as positive against all others to compute class-specific precision and recall. Macro-averaged results are reported to reflect overall performance across classes. All metrics are computed on the held-out test set.

## 3. Results

### 3.1. Overall Performance

To comprehensively evaluate the effectiveness of E-GhostNetV3, we compare it with representative lightweight models, including GhostNetV3 (1.3×), MobileNetV2 [14], MobileNetV3-Large [15], MobileNetV3-Small [15], EfficientNet-B0 [16], MobileNetV4-Conv-S [17], and RepViT-M1.0 [18]. In addition, we also include InceptionV3 [19] and DenseNet-121 [20], which have larger parameter sizes, as performance upper-bound references between lightweight models and larger-capacity models, to more comprehensively position the performance level of E-GhostNetV3. All models were trained using the same training configuration and dataset split. For each run of each model, the checkpoint with the highest validation accuracy was selected for evaluation on the test set.

Table 5 reports the performance comparison of all models. E-GhostNetV3 achieves the highest average Top-1 accuracy of 89.61% on the eggplant fruit disease dataset, outperforming the original GhostNetV3 (87.07%) by 2.54 percentage points.

To further assess the statistical reliability of the performance gain, a paired *t*-test was conducted on the five repeated runs. The difference was defined as GhostNetV3 minus E-GhostNetV3. The test yielded a t-statistic of −3.57 with 4 degrees of freedom, an exact *p*-value of 0.02336, and a Cohen’s d effect size of 1.60, indicating a statistically significant and practically meaningful improvement over the baseline.

To facilitate a fair and controlled comparison, all models, including the baseline and the proposed E-GhostNetV3, were trained under the same experimental settings, without introducing specialized imbalance-handling techniques such as Focal Loss or class weighting. We used a stratified 8:1:1 split and standard data augmentation, with Mixup set to 0.1 and CutMix set to 0.3. By keeping the loss functions and sampling strategies consistent across models, this experimental setup helps reduce potential confounding effects from differences in loss functions and sampling strategies, thereby providing a more consistent basis for examining the effects of architectural changes.

E-GhostNetV3 achieves a mean accuracy of 89.61% with 1.81 M parameters. Its default THOP estimate is 116.60 M MACs, compared with 276.52 M for the original GhostNetV3, 55.52 M for MobileNetV3-Small, and 2833.37 M for DenseNet-121. These values describe the operations covered by the adopted counting rules and should not be interpreted as complete operation counts or evidence of faster inference. The results support improved recognition accuracy with a compact parameter count, while practical computational efficiency remains to be evaluated on target hardware.

### 3.2. Ablation Study

To evaluate the contribution of the backbone reconfiguration and the proposed modules, we progressively evaluate backbone reconfiguration, SE removal, WCD-Down, and DySCA under controlled configurations, as summarized in Table 6.

The original GhostNetV3 (1.3×) achieves an accuracy of 87.07% with 7.67 M parameters and a default THOP estimate of 276.52 M MACs. After backbone reconfiguration while retaining the SE modules, the parameter count is substantially reduced to 2.18 M and the default THOP estimate decreases to 99.13 M MACs, while the accuracy decreases to 84.64%. This result quantifies the performance trade-off introduced by the backbone reconfiguration. When the SE modules are further removed from the reconstructed backbone, the parameter count decreases to 1.80 M and the accuracy increases to 86.63%. Thus, compared with the reconstructed backbone with SE, removing SE reduces the parameter count by approximately 0.38 M while improving accuracy by 1.99 percentage points. These results indicate that the effects of backbone compression and SE removal should be considered separately.

We next evaluate the contribution of WCD-Down under both SE-retained and SE-free configurations. When WCD-Down is added to the reconstructed backbone with SE, the accuracy increases from 84.64% to 87.41%, corresponding to a 2.77 percentage point improvement, with only a marginal increase in parameters and the default THOP estimate. Under the SE-free configuration, adding WCD-Down further improves the accuracy from 86.63% to 87.85%, corresponding to a 1.22 percentage point improvement, while the parameter count remains nearly unchanged and the default THOP estimate increases slightly. These comparisons show higher mean accuracy with WCD-Down under both SE-retained and SE-free configurations.

WCD-Down is designed to combine Haar-based multiband representations with learnable spatial features. The controlled comparisons show higher mean accuracy with WCD-Down under both SE-retained and SE-free configurations; however, these results alone do not establish which internal mechanism accounts for the observed differences.

For the attention mechanism, we compare DSA and DySCA using the same reconstructed SE-free backbone. DSA achieves an accuracy of 86.19%, whereas DySCA applied at Stages 1 and 2 achieves 88.29%. Thus, DySCA provides a 2.10 percentage point improvement over DSA under the same backbone configuration. Compared with the reconstructed SE-free backbone without an additional attention module (86.63%), DySCA improves accuracy by 1.66 percentage points. When DySCA is extended to all stages (Stages 1–4), the accuracy decreases to 86.41%, compared with 88.29% when it is applied only at Stages 1 and 2. The earlier stages retain relatively higher spatial resolution and fine-grained local information, whereas repeated downsampling in deeper stages progressively reduces spatial resolution and changes the characteristics of the feature representations. Therefore, extending DySCA to deeper stages does not necessarily provide additional performance benefits. The higher accuracy obtained with DySCA at Stages 1 and 2 empirically supports the selected placement; however, these ablation results alone do not directly establish the underlying feature-level mechanism responsible for this performance difference.

Finally, combining WCD-Down at the Stem stage with DySCA at Stages 1 and 2 yields the proposed E-GhostNetV3, achieving 89.61% accuracy with 1.81 M parameters and a default THOP estimate of 116.60 M MACs. Relative to the reconstructed SE-free backbone, the complete model improves accuracy from 86.63% to 89.61%, corresponding to a 2.98 percentage point improvement. Relative to the original GhostNetV3, E-GhostNetV3 achieves a 2.54 percentage point higher mean accuracy while reducing the reported parameter count by approximately 76.40%. Compared with the reconstructed SE-free backbone with WCD-Down alone, adding DySCA improves accuracy by a further 1.76 percentage points, with a modest increase in parameters and the default THOP estimate. Overall, the controlled ablation results help distinguish, to some extent, the performance contributions of SE removal, WCD-Down, and DySCA under the evaluated configurations, but do not directly establish the internal mechanisms underlying the observed differences.

### 3.3. Confusion Matrix Analysis

**Figure 5:**
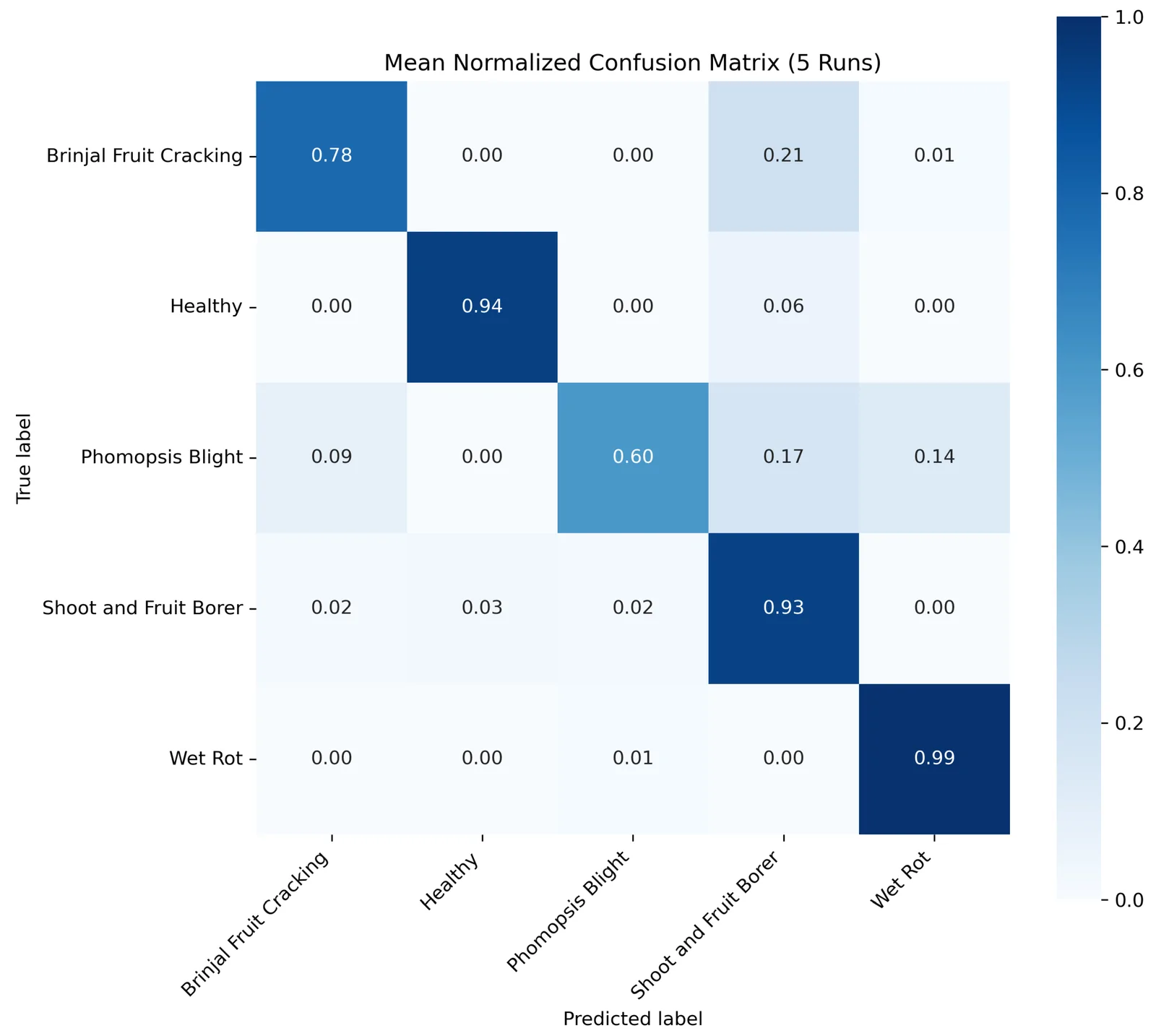
Mean normalized confusion matrix across five runs.

Figure 5 presents the mean normalized confusion matrix of E-GhostNetV3 across five independent runs on the test set, while Table 7 summarizes the mean precision, recall, and F1-score for each class across the same five runs. For each run, the confusion matrix was normalized row-wise, and the resulting normalized matrices were then averaged element-wise. Accordingly, the diagonal elements in Figure 5 represent the mean class-specific recall across the five runs and correspond to the recall values reported in Table 7. Overall, E-GhostNetV3 achieves a weighted-average precision of 89.62%, a weighted-average recall of 89.61%, and a weighted-average F1-score of 89.28%. The predominance of diagonal entries in the mean normalized confusion matrix further indicates that most samples are correctly classified across the repeated experiments.

The model performs relatively less effectively on Phomopsis Blight and Brinjal Fruit Cracking. Phomopsis Blight achieves a mean recall of 60.00%, which is substantially lower than its mean precision of 85.62%, indicating that a considerable proportion of samples belonging to this class are misclassified as other classes. This makes Phomopsis Blight the most challenging class to identify among the five categories. The mean confusion matrix shows that Phomopsis Blight is most frequently confused with Shoot and Fruit Borer, with approximately 17% of Phomopsis Blight samples being classified as Shoot and Fruit Borer. Additional confusion is observed with Wet Rot and Brinjal Fruit Cracking, accounting for approximately 14% and 9% of the samples, respectively. For Phomopsis Blight, the mean recall of 60.00% corresponds to an average of 9.6 correctly classified images and 6.4 misclassified images per run. The 95% confidence interval for its mean recall is 49.59–70.41%. With only 16 test images, a single classification error changes the per-run recall by 6.25 percentage points. This small test sample makes the estimate sensitive to individual errors, but does not by itself explain the low recall.

Brinjal Fruit Cracking achieves a mean precision of 84.99% and a mean recall of 78.00%. Its relatively lower recall indicates that a proportion of Brinjal Fruit Cracking samples are misclassified as other classes. The mean confusion matrix shows that the primary confusion direction is toward Shoot and Fruit Borer, with approximately 21% of Brinjal Fruit Cracking samples being classified as Shoot and Fruit Borer. Conversely, the precision of Brinjal Fruit Cracking is also affected by samples from other categories being assigned to this class, indicating a degree of bidirectional confusion. From a visual recognition perspective, Brinjal Fruit Cracking manifests as physical rupture of the fruit skin, and the resulting damaged regions may exhibit irregular morphology and color variations that can partially resemble insect-related damage. Such visual similarities may contribute to the observed confusion between these two categories.

The Healthy class achieves a mean precision of 96.02% and a mean recall of 94.12%. The higher precision than recall indicates that predictions assigned to the Healthy class are generally reliable, while some Healthy samples are classified as other categories. The mean confusion matrix shows that approximately 6% of Healthy samples are classified as Shoot and Fruit Borer, while the remaining misclassification rates are relatively small. However, this difference alone does not establish a systematic tendency to classify ambiguous diseased samples as Healthy, and therefore should not be interpreted as evidence of a specific omission mechanism.

Wet Rot exhibits relatively high recall values of 99.09%. In the mean confusion matrix, most samples from the classes remain on the diagonal, indicating relatively stable recognition across the five independent runs. In particular, Wet Rot shows the highest mean recall among the five classes, with only approximately 1% of its samples being misclassified as Phomopsis Blight.

Despite the above confusions, E-GhostNetV3 achieves a mean F1-score above 70% for all five classes, with a macro-averaged F1-score of 86.22%. These results indicate that the model maintains relatively strong overall classification performance across the five classes despite the imbalanced class distribution.

## 4. Discussion

In real agricultural production, accurately identifying eggplant fruit diseases faces multiple challenges. On the one hand, there is significant inter-class similarity among different diseases; for example, Wet Rot and Phomopsis Blight may exhibit similar color changes and textural characteristics at the late stage of decay. On the other hand, there is notable intra-class variation within the same disease class, as lesion morphology, size, and color continuously change with fruit maturity and disease progression. In addition, natural field images are subject to interference from illumination variations, foliage occlusion, and complex backgrounds, which may reduce the discriminative capability of lightweight convolutional networks.

To address these challenges, the proposed E-GhostNetV3 improves recognition performance through two core module enhancements. First, the WCD-Down module is introduced at the downsampling stage to enhance feature representation from complementary frequency-domain and spatial-domain perspectives. Unlike Finder et al. [11], who integrate wavelet transform into convolutional layers (WTConv), this work adopts the wavelet transform as an independent preprocessing branch running in parallel with the standard convolution branch, and fuses the resulting features through convolution. This design allows the wavelet branch to provide explicit structural and multiband information while retaining the learnable spatial feature extraction capability of the convolutional branch. Second, the DySCA module is deployed after Stages 1 and 2 of the shallow network to perform adaptive reweighting of intermediate feature maps along both spatial and channel dimensions, with the design intention of strengthening the network’s response to informative spatial locations and discriminative feature channels. The two modules are designed to complement each other: WCD-Down aims to alleviate detail loss during downsampling and provide richer structural information for subsequent feature extraction, while DySCA is intended to enhance the representation of fine-grained disease characteristics through spatial and channel reweighting.

The controlled ablation results in Table 6 compare configurations differing in SE removal, WCD-Down, and DySCA. Removing SE from the reconstructed backbone improves the accuracy from 84.64% to 86.63%, corresponding to a 1.99 percentage point improvement, while also reducing the parameter count from 2.18 M to 1.80 M. When WCD-Down is added to the SE-free reconstructed backbone, the accuracy further increases from 86.63% to 87.85%, corresponding to a 1.22 percentage point improvement. Under the SE-retained configuration, WCD-Down also improves the accuracy from 84.64% to 87.41%, with higher mean accuracy observed under both SE settings. DySCA applied at Stages 1 and 2 further improves the accuracy from 87.85% to 89.61% when combined with WCD-Down, corresponding to a 1.76 percentage point improvement. Overall, relative to the reconstructed SE-free backbone, the complete E-GhostNetV3 improves accuracy from 86.63% to 89.61%, corresponding to a 2.98 percentage point improvement. These controlled comparisons document the mean accuracy differences associated with the evaluated architectural changes.

Although the internal ablation experiments provide evidence supporting the proposed modules under the evaluated settings, there remains a noticeable accuracy gap when comparing our method with existing studies that employ pre-trained weights. Notably, compared with the 97.98% accuracy reported by Nath et al. [9] using ImageNet pre-trained weights, our 89.61% accuracy achieved under training-from-scratch conditions is inferior. This difference should be interpreted in the context of the distinct training paradigms. Their study relies on ImageNet pre-trained weights for transfer learning or fine-tuning, whereas all experiments in this study are consistently trained from scratch without external pre-trained weights. Nath et al. [9] also reported substantial performance degradation for lightweight models under the same training-from-scratch condition. Therefore, the contribution of this work is not to outperform transfer-learning-based results, but to demonstrate that, under a training-from-scratch setting, architectural modifications based on WCD-Down and DySCA can improve the recognition performance of a lightweight backbone while retaining a compact parameter count. This result highlights the potential of architecture-level improvements when external pre-trained weights are not used.

From an application perspective, training from scratch can also be relevant in scenarios where external pre-trained models or large-scale auxiliary datasets are unavailable or undesirable. In real agricultural applications, field images may differ substantially from general-purpose datasets such as ImageNet in terms of background, illumination, object appearance, and disease characteristics. Accordingly, the effectiveness of transferred representations may vary with the degree of domain difference between the source and target data. The approach investigated in this study therefore provides an alternative pathway for improving lightweight disease recognition through architectural design without relying on external pre-trained weights. This characteristic may be beneficial for application scenarios that prioritize training-data independence, model compactness, and computational efficiency.

However, the proposed method also has limitations. First, the current experiments rely primarily on a single eggplant fruit disease dataset, with limited geographic coverage and cultivar diversity, and the class distribution is not fully balanced, with fewer samples available for Phomopsis Blight. Although stratified splitting and identical training settings were used for all models, the dataset was divided at the image level because image-level metadata linking images to specific fruits, plants, acquisition sessions, or locations are not available. Therefore, the current split does not explicitly guarantee that visually correlated images from the same fruit, plant, acquisition session, or field scene are separated across the training, validation, and test sets. This may introduce some degree of sample correlation and could lead to optimistic estimates of generalization performance. Accordingly, the reported results should be interpreted as performance under an image-level stratified split rather than as evidence of strict cross-plant, cross-fruit, cross-session, or cross-location generalization. Although the dataset contains field-captured images, robustness to specific variations in illumination, occlusion, and background was not evaluated separately. The reported results therefore should not be interpreted as evidence of robustness to these individual factors. The relatively small number of samples in the Phomopsis Blight class may also affect the robustness of class-specific performance estimates. Specialized class-imbalance strategies, such as Focal Loss or class-weighted loss, were not investigated in the present study and could be explored in future work. Second, all experiments depend solely on RGB images and do not exploit the rich spectral information available from multispectral or hyperspectral sensors. Third, the evaluation is currently conducted offline on GPUs only, without deployment validation on actual edge devices such as Jetson modules or smartphones. Fourth, computational complexity was estimated using the default rules of THOP 0.1.1 without custom counting rules. Functional dynamic convolutions and other unsupported operations are not fully covered. Consequently, the reported MACs estimates do not establish complete computational costs, and differences in counting coverage may affect comparisons across architectures.

Future work will extend the evaluation to a wider range of multi-site field datasets and different eggplant varieties, together with external validation using images acquired with different imaging devices. Where appropriate acquisition metadata are available, more stringent plant-, fruit-, acquisition-session-, or geographic-location-level evaluation will also be investigated. We will also explore multimodal sensor fusion strategies, conduct deployment experiments and inference optimization on real edge devices, and integrate domain generalization techniques to investigate cross-scenario robustness.

## 5. Conclusions

Experiments on the BrinjalFruitX dataset, which contains 1823 real field-captured images, demonstrate that E-GhostNetV3 achieves a Top-1 accuracy of 89.61% under training-from-scratch conditions, with 1.81 M parameters. This represents a 2.54 percentage point improvement over the GhostNetV3 baseline (87.07%). The default THOP estimate is 116.60 M MACs, subject to the operation-coverage limitations described above. Controlled ablation studies provide evidence supporting the proposed modules through comparisons of mean accuracy across the evaluated configurations.

Unlike the benchmarking study of Nath et al. [9], which aimed to select the best performer from off-the-shelf models using ImageNet pre-training, this work focuses on improving the performance of lightweight models through modular architectural innovations without pre-training. The experimental results demonstrate that this architecture-level improvement strategy is effective under the evaluated training-from-scratch setting. The compact parameter count motivates future evaluation on edge devices. However, inference latency, throughput, memory usage, and energy consumption have not been measured on target devices. On-device validation therefore remains future work, and edge deployment should be regarded as a potential application rather than a demonstrated capability.

Future work will extend the evaluation to a wider range of multi-site field datasets, different eggplant varieties, and external validation using images acquired with different imaging devices. Where appropriate acquisition metadata are available, more stringent plant-, fruit-, acquisition-session-, or geographic-location-level evaluation will also be investigated. We will also explore multimodal sensor fusion strategies, conduct deployment experiments on actual edge devices, and investigate model compression and domain generalization techniques to further enhance practical value.

## Figures and Tables

**Table 1:** Sample distribution of the dataset.

Class	Sample Count	Proportion
Shoot and Fruit Borer	725	39.77%
Healthy	514	28.20%
Wet Rot	223	12.23%
Brinjal Fruit Cracking	200	10.97%
Phomopsis Blight	161	8.83%

Note: Proportions in the table are subject to rounding, and the sum may not equal 100%.

**Table 2:** Architectural comparison between the original GhostNetV3 and the reconstructed SE-free backbone.

Stage	Original GhostNetV3 (w=1.3)	Reconstructed GhostNetV3 (w=1.0)
Stage 1	1 block; k=3; t/c=16/16; s=1; SE: No	Same
Stage 2	2 blocks; k=3; t/c=48/24, 72/24; s=2,1 SE: No	Same
Stage 3	2 blocks; k=5; t/c=72/40, 120/40; s=2,1 SE: Yes	2 blocks; same t/c and strides; SE: No
Stage 4	6 blocks; k=3; t/c=240/80, 200/80, 184/80, 184/80, 480/112, 672/112; s=2,1,1,1,1,1 SE: mixed	4 blocks; k=3; t/c=240/80, 200/80, 184/80, 184/80; s=2,1,1,1 SE: No
Stage 5	5 blocks; k=5; t/c=672/160, 960/160, 960/160, 960/160, 960/160; s=2,1,1,1,1 SE: mixed	3 blocks; k=5; t/c=256/160, 512/160, 768/160; s=2,1,1 SE: No

Note: *w* denotes the model width. *k* denotes the depthwise convolution kernel size; *t* denotes the intermediate channel dimension; *c* denotes the output channel dimension; *s* denotes the stride. “SE: Yes” indicates an SE ratio of 0.25, whereas “SE: No” indicates that the SE module is removed.

**Table 3:** Structured comparison of WCD-Down/DySCA with representative modules (conceptual positioning).

Module	Core Mechanism	Placement	Advantages	Limitations
Standard Convolutional Downsampling	Strided convolution	Stem stage	Simple and efficient	May lose or blur fine-grained spatial details during resolution reduction
Haar Wavelet Extraction	Frequency decomposition	Stem stage	Preserves low-frequency structural contours and high-frequency details	No learnable parameters, lacks adaptability
SE [13]	Global average pooling followed by fully connected layers to obtain attention scores for channel reweighting	Inside GhostBottleneck	Lightweight, captures global channel dependencies	Channel-only, ignores spatial information
DSA	Dynamically generates spatial convolution kernels and convolves them with channel-wise mean results to obtain spatial attention weights	Stages 1 and 2	Adaptive spatial modulation, focuses on target regions	Uses only mean statistics, lacks perception of local extremes and dispersion
WCD-Down (Ours)	Wavelet decomposition and standard convolution	Stem stage	Combines spatial and frequency-domain representations	Slightly higher parameter count than plain convolution
DySCA (Ours)	Dynamically generates spatial and channel attention; spatial attention fuses mean, max, and standard deviation statistics	Stages 1 and 2	Dynamic spatial-channel collaboration, comprehensive perception of spatial distribution via multiple statistics	Additional operations for dynamic kernel generation and spatial–channel attention

Note: This table provides a conceptual positioning and is not intended to be exhaustive.

**Table 4:** Unified training configuration for baseline and E-GhostNetV3.

Parameter	Baseline	E-GhostNetV3	Explanation
Input shape	3×224×224	3×224×224	Uniform input resolution
Batch size	32	32	Uniform batch size
Number of epochs	150	150	Training epochs remain consistent
Optimizer	AdamW	AdamW	Unified optimization algorithm
Initial learning rate	0.0015	0.0015	Consistent learning rate
Weight decay	$2\times 10^{-5}$	$2\times 10^{-5}$	Weights remain consistent
Data augmentation	horizontal flip (0.5), color disturbance (0.4)	Same as baseline	Basic enhancements remain consistent
Mixup, CutMix	0.1, 0.3	0.1, 0.3	Identical strong augmentation for fairness
Mixed precision	✓	✓	GPU-side training acceleration (AMP)

**Table 5:** Performance comparison of E-GhostNetV3 with representative lightweight models on the eggplant fruit disease dataset.

Model	Acc ± 95CI (%)	Prec ± 95CI (%)	Recall ± 95CI (%)	F1 ± 95CI (%)	ΔAcc ± 95CI (%)	MACs (M)	Params (M)
GhostNetV3 (1.3×)	87.07±1.43	86.00±2.53	82.15±2.14	83.13±1.95	-	276.52	7.67
MobileNetV2-100	77.79±1.32	76.89±3.71	69.15±2.38	69.05±4.20	−9.28±0.89	299.56	2.20
MobileNetV3-Large	68.51±15.26	61.48±27.95	54.56±23.68	52.41±26.96	−18.56±14.76	215.36	4.18
MobileNetV3-Small	77.79±9.91	77.00±10.58	70.44±16.37	70.76±17.77	−9.28±10.78	55.52	1.51
EfficientNet-B0	83.10±1.24	81.95±0.82	78.25±2.57	78.99±2.01	−3.98±1.56	384.61	3.97
InceptionV3	85.30±1.04	84.07±1.38	80.77±2.16	81.51±1.87	−1.77±1.96	2835.94	21.76
DenseNet-121	87.96±0.76	85.22±2.66	82.94±1.22	83.90±1.66	0.88±0.92	2833.37	6.88
MobileNetV4-Conv-S	76.13±1.13	75.25±2.68	66.34±2.42	66.87±2.73	−10.94±1.32	184.77	2.47
RepViT-M1.0	83.76±2.41	80.36±3.91	79.71±3.44	79.75±3.46	−3.32±2.74	846.29	4.72
E-GhostNetV3 (Our)	89.61±0.89	88.80±1.69	84.91±1.87	86.22±1.94	2.54±1.97	116.60	1.81

Note: Precision, Recall, and F1-score are macro-averaged. MACs are estimated using the default rules of THOP 0.1.1 without custom counting rules. Unsupported operations are not fully covered, and counting coverage may differ across models. The estimates do not indicate measured hardware performance. The counting rules for MACs in Table 6 are the same. MobileNetV3-Small and MobileNetV3-Large exhibit significant performance fluctuation when trained from scratch, with test accuracies varying between 60.22% and 85.64% across five runs, resulting in wide confidence intervals. Acc ± 95% CI is calculated based on five repeated experiments using Student’s t-distribution (df = 4); the same applies to Table 6.

**Table 6:** Ablation study of backbone reconfiguration and modules on the BrinjalFruitX dataset.

Model	Backbone Reconfiguration	SE	WCD-Down	DSA	DySCA	Placement	Params (K)	MACs (M)	Acc ± 95CI (%)	ΔAcc ± 95CI (%)
GhostNetV3 (1.3×)	-	-	-	-	-	-	7673.70	276.52	87.07±1.43	-
GhostNetV3 + Backbone + SE	✓	✓	-	-	-	-	2179.55	99.13	84.64±1.84	−2.43 ± 1.65
GhostNetV3 + Backbone − SE	✓	-	-	-	-	-	1803.99	98.48	86.63±1.91	−0.44 ± 1.71
GhostNetV3 + Backbone + SE + WCD-Down	✓	✓	✓	-	-	Stem	2179.71	101.64	87.41±1.90	+0.33 ± 3.09
GhostNetV3 + Backbone − SE + WCD-Down	✓	-	✓	-	-	Stem	1804.16	100.99	87.85±1.08	+0.77 ± 1.91
GhostNetV3 + Backbone − SE + DSA	✓	-	-	✓	-	Stage 1, 2	1805.91	98.76	86.19±1.75	−0.88 ± 2.25
GhostNetV3 + Backbone − SE + DySCA	✓	-	-	-	✓	Stage 1, 2	1809.53	114.09	88.29±0.89	+1.21 ± 0.57
GhostNetV3 + Backbone − SE + DySCA (All Stage)	✓	-	-	-	✓	Stage 1, 2, 3, 4	1853.55	121.73	86.41±2.60	−0.67 ± 3.15
E-GhostNetV3 (Our)	✓	-	✓	-	✓	Stem, Stage 1, 2	1809.70	116.60	89.61±0.89	+2.54 ± 1.97

Note: Params are reported in thousands (K) to clearly show the parameter changes introduced by each module, as the increments are small and the values are close to each other (approximately 1.81 M) when expressed in millions. The parameter counts reported in the main text are rounded to two decimal places in millions (M). ΔAcc denotes the mean accuracy of each configuration minus that of the original GhostNetV3 baseline and is expressed in percentage points (pp). The accompanying interval is the 95% confidence interval for this mean difference.

**Table 7:** Mean per-class performance and counts across five independent runs.

Class	Precision (%)	Recall ± 95% CI (%)	F1-Score (%)	Mean TP	Mean FN	Support
Brinjal Fruit Cracking	84.99	78.00±7.08	81.18	15.60	4.40	20
Healthy	96.02	94.12±1.72	95.05	48.00	3.00	51
Phomopsis Blight	85.62	60.00±10.41	70.37	9.60	6.40	16
Shoot and Fruit Borer	87.06	93.33±1.44	90.08	67.20	4.80	72
Wet Rot	90.28	99.09±2.52	94.41	21.80	0.20	22
Macro-average	88.79	84.91	86.22	–	–	181
Weighted-average	89.62	89.61	89.28	–	–	181

Note: TP and FN denote correctly classified samples and samples misclassified as other classes, respectively. Mean TP and Mean FN are averaged across five runs; support denotes the number of test samples per run. Counts were reconstructed from the rounded row-normalized confusion matrices using the known class supports. Recall confidence intervals were calculated from the five per-run recall values using Student’s t-distribution with four degrees of freedom. Values following ± are confidence-interval half-widths in percentage points. These intervals describe between-run variability on the test set and do not fully capture uncertainty due to limited test sampling. The unbounded t interval for Wet Rot extends above 100%, reflecting a limitation of this approximation. Confidence intervals are reported for individual classes only. Minor discrepancies between tables may arise because the main-table summary statistics were calculated from per-run values rounded to two decimal places.

## Data Availability

The data used in this study are publicly available. The BrinjalFruitX: A Field-Collected Image Dataset for Machine Learning and Deep Learning-Based Disease Identification in Brinjal Fruits dataset can be accessed at https://data.mendeley.com/datasets/ngc58fsxgd/1 (accessed on 29 August 2026). This dataset is used for plant disease classification and model evaluation.
